# Catastrophic health expenditures: a disproportionate risk in uninsured ethnic minorities with diabetes

**DOI:** 10.1186/s13561-024-00486-7

**Published:** 2024-03-06

**Authors:** Sebastian Linde, Leonard E. Egede

**Affiliations:** 1 Department of Health Policy & Management, Texas A&M School of Public Health, 212 Adriance Lab Rd, College Station, Texas, TX 77843 USA; 2 Department of Medicine, Division of General Internal Medicine, Medical College of Wisconsin, 8701 Watertown Plank Rd., Milwaukee, WI 53226-3596 USA; 3 Center for the Advancing Population Sciences, Medical College of Wisconsin, Milwaukee, WI USA

**Keywords:** Catastrophic Health Expenditures, Chargemaster Prices, Diabetes, Uninsured, Racial Disparities, Structural Inequity, Structural Racism

## Abstract

**Background:**

Chargemaster prices are the list prices that providers and health systems assign to each of their medical services in the US. These charges are often several factors of magnitude higher than those extended to individuals with either private or public insurance, however, these list prices are billed in full to uninsured patients, putting them at increased risk of catastrophic health expenditures (CHE). The objective of this study was to examine the risk of CHE across insurance status, diabetes diagnosis and to examine disparity gaps across race/ethnicity.

**Methods:**

We perform a retrospective observational study on a nationally representative cohort of adult patients from the Medical Expenditure Panel Survey for the years 2002–2017. Using logistic regression models we estimate the risk of CHE across insurance status, diabetes diagnosis and explore disparity gaps across race/ethnicity.

**Results:**

Our fully adjusted results show that the relative odds of having CHE if uninsured is 5.9 (*p* < 0.01) compared to if insured, and 1.1 (*p* < 0.01) for patients with a diabetes diagnosis (compared to those without one). We note significant interactions between insurance status and diabetes diagnosis, with uninsured patients with a diabetes diagnosis being 9.5 times (*p* < 0.01) more likely to experience CHE than insured patients without a diabetes diagnosis. In terms of racial/ethnic disparities, we find that among the uninsured, non-Hispanic blacks are 13% (*p* < 0.05), and Hispanics 14.2% (*p* < 0.05), more likely to experience CHE than non-Hispanic whites. Among uninsured patients with diabetes, we further find that Hispanic patients are 39.3% (*p* < 0.05) more likely to have CHE than non-Hispanic white patients.

**Conclusions:**

Our findings indicate that uninsured patients with diabetes are at significantly elevated risks for CHE. These risks are further found to be disproportionately higher among uninsured racial/ethnic minorities, suggesting that CHE may present a channel through which structural economic and health disparities are perpetuated.

**Supplementary Information:**

The online version contains supplementary material available at 10.1186/s13561-024-00486-7.

## Introduction

Prior work has identified medical bills as a primary cause of personal bankruptcies in the U.S. [25–28, 46], and more recent national surveys have further highlighted the prevalence of these adverse events among patients [23]. While the effect of medical bills on personal financial hardship is experienced by both insured and uninsured patients, the risk of medical bills resulting in catastrophic health expenditures (that is, billed amounts exceeding 40% of an individual’s post subsistence income) (CHE) is likely to be higher for uninsured patients who are billed based on providers’ chargemaster rates [4, 9, 10]. Chargemaster rates are list prices that providers and health systems assign to each of their medical services, and these list prices are often several factors of magnitude higher than those extended to individuals with either private or public insurance [4, 9, 10, 13, 14].

Additionally, CHE are likely to be higher among individuals with chronic health conditions such as diabetes, with these patients experiencing well documented elevated expenditures [15, 41, 53]. As such, we hypothesize that the risk of CHE is significantly higher among uninsured individuals who have a diabetes diagnosis. Furthermore, based on prior work showing that racial/ethnic minorities tend to be overrepresented within the uninsured patient population [7], and among those with a diabetes diagnosis [16], we also hypothesize that there may exist racial/ethnic disparities within CHE risks.

While past work has examined CHE related to a few selected medical conditions and medical events [30, 42, 46, 54], there has been no broad exploration of the uninsured and patients with diabetes, despite the potential high risk for experiencing a CHE within this population. As such, a contribution of our study to prior literature is the examination of CHE risk within these populations within the US health care context. Second, prior work has focused solely on expenditures instead of the amounts that are billed to the patient with expectations for payment. The present study seeks to address this issue by focusing analysis on charges incurred by uninsured patients as opposed to payments made. This focus is important as it better captures the required payments for uninsured patients within the US market, but it is also important from a policy intervention perspective as legislation targeting maximum allowable chargemaster markups above cost may be legislatively feasible, as well as legislation ensuring application of standard contract law to chargemaster billing cases [44]. Such considerations are of policy relevance as recent work has noted significant growth and variation within chargemaster markups above costs over the past two to three decades within the US hospital market [35]. With this noted, our study contributes to prior literature by focusing on these chargemaster (billed) amounts, and in so doing, it aims to provide further input to policy discussions surrounding chargemaster levels within the US health care market. Lastly, within prior work, there has been little discussion of the extent to which racial and ethnic minorities may be particularly at risk for CHE. An examination of racial/ethnic disparities related to CHE exposure are particularly important within the US health care context as recent research has indicated significant relationships between historic structural racist laws and present-day structural inequities and health disparities [11, 12, 20, 34, 36]. If CHE risk is significantly higher among racial/ethnic minority populations, then policies aimed at reducing chargemaster prices may further act to ameliorate structural inequities in CHE exposure. As such, in studying racial/ethnic disparities in CHE risk, our study seeks to contribute to the literature on CHE and to policy discussions surrounding how structural racial/ethnic inequities may be addressed within the US health care setting.

In summary, the present study seeks to address existing knowledge gaps within the CHE literature by focusing analysis on the charges billed to patients and the resulting risk of experiencing a CHE among patients who are uninsured and have a diabetes diagnosis, while also examining disparity gaps within CHE risk across different racial and ethnic groups. If CHE disparity gaps persist across race/ethnicity, even after adjusting for patient differences in factors that drive patient utilization of health care services, then this may present an important channel through which economic and health disparities may be perpetuated, which in turn may provide an opportunity to identify policies and strategies to reduce health disparities across race and ethnicity.

## Overview of US healthcare payment system

The US healthcare system is a mixed system that can broadly be characterized by three sets of primary payors – private insurers, public insurers, and the uninsured – who all face different payment modalities [29]. Pertaining to private insurers, these negotiate their covered patients’ rates (i.e., their payments) with health care providers directly. As such, rates between private payors and health care providers are a function of the relative bargaining power of each party. Recent findings indicate that these payment rates are commonly either negotiated off of the chargemaster rates (this is more common within markets where providers have a strong bargaining position relative to private payors), or as a markup above what public payors such as Medicare pay (this is more common in markets where providers have a weaker bargaining position) [19]. Public payors such as Medicare, who provide coverage for primarily the older US population (aged 65 and older), and Medicaid, who provide coverage primarily to enrolled low-income individuals (note: eligibility criterions varying across states), on the other hand, set their own reimbursement schedules that are primarily based on cost-based capitated reimbursement, or fee-for-service payments [16, 29, 39]. Uninsured patients, however, do not benefit from any of these negotiated or set payment rates, and are instead billed the much higher chargemaster prices in full. Chargemaster prices represent significant markups above what both private and public payors pay, and related to the research objective of our study, this feature puts uninsured patients at an increased risk of CHE within the US health care system [4, 13, 14].

## Methods

### Data sources and study sample

We retrospectively examine a sample of 256,280 individuals with medical charges, aged 18 years and older, and with a race/ethnicity of non-Hispanic White (NHW), non-Hispanic Black (NHB) or Hispanic, for the 2002 – 2017 period using the full-year household consolidated data files of the Medical Expenditure Panel Survey (MEPS) [1]. As such, the unit of observation is at the adult individual-year level, and choice of race/ethnicity inclusion was done to ensure sufficient samples. MEPS is an annual national survey that derives estimates of healthcare utilization, health status, and health insurance coverage. The MEPS survey employs a stratified sampling design to provide a nationally representative sample of the civilian noninstitutionalized U.S. population [2].

### Study variables

## Outcome variable

Our outcome measure of catastrophic health expenditures (CHE) is defined as any out-of-pocket (i.e., total) health charges that exceed 40% of a patient’s post subsistence income (i.e., income after adjustment for food expenditures) [30, 42, 46, 54]. This CHE measure is a binary indicator variable that takes the value 1 (if out-of-pocket charges exceed 40% of post subsistence income) and 0 otherwise. We further note that this measure is defined at the individual level, and that it depends on four factors – the patient’s individual income, the amount they spend on food, their out-of-pocket charges, and the percentage cutoff for charges relative to the post subsistence income that is used.

For our income measure, we use the patient’s total annual income from the MEPS, which we inflation adjust into 2017 dollars using the Consumer Price Index (CPI) (BLS 2017). In order to obtain the individual post subsistence income, we then subtract the estimated national amounts dedicated to annual food spending. This adjustment is done based on the Bureau of Labor Statistics food expenditure estimates for 2017, and these adjustments are customized based on the individuals’ income decile (BLS 2019). Our out-of-pocket charges are based on total charges (across all medical events) for uninsured patients, and based on the self-pay (out-of-pocket) portion of charges for insured patients. Lastly, we follow prior work and base our CHE threshold at a 40% cutoff of post subsistence income [30, 46, 54].

## Primary independent variables

Our primary independent variables of interest consist of patient insurance coverage, diabetes diagnosis, and race/ethnicity variables. For race/ethnicity, we include NHW, NHB, and Hispanic patient indicators. A patient is coded as uninsured if they report having been uninsured for the full calendar year, and their diabetes status is based on whether they have a diagnosis of diabetes.

## Covariates

One of the challenges in understanding the risk of experiencing a CHE among patients may stem from the patient’s choice to receive or not receive medical care. Because patients without insurance are aware of their insurance status and financial situation, we need to be able to account for factors that also influence patients’ desire to seek medical care. To account for this selection problem, we employ a rich set of controls that build on the health care utilization framework of Andersen and Newman, along with additional controls for the medical service setting [3].

The Andersen and Newman Framework of Healthcare Utilization categorizes factors that influence utilization as: predisposing, enabling and need based [3, 47]. Predisposing factors are characteristics that exist before the onset of illness and can be associated with different patterns of service utilization. These factors include age, sex, marriage status, and family size within our data. Enabling factors capture the resources that are available to an individual, and which allow them to obtain medical care. To capture this dimension of enabling factors, our CHE measure is adjusted for post subsistence income, and we further include an indicator for whether the patient is employed, and whether they have earned a bachelors college degree (or higher). Need factors are the perceived or evaluated presence of an illness that would provide the patient with a reason for seeking medical care. These factors are captured using information on patient diagnosis of high blood pressure, coronary heart disease, stroke, emphysema and arthritis. Additionally, we also include indicators for whether the patient reported needing help with activities of daily living (ADL) and/or instrumental activities of daily living (IADL). Patients’ self-reported physical health and mental health scores are converted into binary indicators with responses of poor or fair health coded as a one, and other responses coded as zero.

Lastly, we also include controls for the type of medical events a patient had. That is, we supplement the controls that drive health care utilization (within the Anderson and Newman Framework) with indicators of whether a patient received any office-based, emergency room, outpatient department, and/or inpatient care.

### Statistical analysis

We utilize logistic regression methods to estimate the relationship between the risk of CHE and our primary independent variables across two sets of analyses – the first looking at CHE disparities across insurance status and diabetes diagnosis; the second analyzing racial/ethnic disparity gaps among the uninsured and diabetes diagnosis sub-populations (i.e. within the populations of potentially elevated CHE risk). To mitigate concerns of bias with our observational study design we take a number of important steps. Firstly, we included controls for the predisposing, enabling and need factors that we believe are important for explaining why an individual may utilize medical care and therethrough be at an increased risk of having CHEs. Additionally, we also control for the type of medical events/care that the patient receives across office-based visits, emergency room visits, outpatient department visits, and inpatient discharges. These controls help ensure that the estimated disparity gaps are not primarily driven by different patient segments receiving care within diverse medical environments/settings. Second, we control for census region and year indicators to account for potential confounding from unobserved geographic and time effects. Third, we also included interaction terms between our primary independent variables (within our first set of analysis) in order to ensure a flexible model specification.

As noted, our first set of analysis focuses on identifying disparity gaps in CHE across insurance coverage and diabetes status, and it also explores whether there exists any significant interaction between these factors. As such, the pooled logistic model specification within our first set of analysis is given by:

1 $$\begin{array}{c}{{\text{log}}}{CHE}_{i}=\alpha +{\tau }_{1}{Uninsured}_{i}+{\tau }_{2}{Diabetes}_{i}+{\tau }_{3}{Uninsured}_{i}*{Diabetes}_{i}\\ +{\varvec{\beta}}{\varvec{X}}+{{\varvec{\phi}}}_{{\varvec{r}}}+{{\varvec{\lambda}}}_{{\varvec{t}}}+{\epsilon }_{i}.\end{array}$$

In Eq. (1), log indicates that this is a logistic model, ${CHE}_{i}$ represents our patient level (binary) outcome measure of CHE, ${Uninsured}_{i}$ captures the insurance status of individual *i*, and ${Diabetes}_{i}$ is an indicator for whether the patient has a diabetes diagnosis. Additionally, ${\varvec{X}}$ is a vector of our covariates (explained above), ${{\varvec{\phi}}}_{{\varvec{r}}}$ captures census region indicators, ${{\varvec{\lambda}}}_{{\varvec{t}}}$ captures year indicators, and $\alpha$, ${\tau }_{1}$, ${\tau }_{2}$, ${\tau }_{3}$, ${{\varvec{\beta}}}$ capture our other model parameters.

Our second set of analysis seeks to examine racial/ethnic disparity gaps within the risk of CHE among the uninsured, diabetes diagnosed, and both uninsured and diabetes diagnosed, sub-populations. This is evaluated using the following pooled logistic model specification:

2 $$\begin{array}{c}{{\text{log}}}{CHE}_{i}=\alpha +{\tau }_{1}{NHB}_{i}+{\tau }_{2}{Hispanic}_{i}+{\tau }_{3}{Uninsured}_{i}+{\tau }_{4}{Diabetes}_{i}\\ +{\varvec{\beta}}{\varvec{X}}+{{\varvec{\phi}}}_{{\varvec{r}}}+{{\varvec{\lambda}}}_{{\varvec{t}}}+{\epsilon }_{i} \end{array}$$

In Eq. (2) ${NHB}_{i}$ is an indicator variable for whether the patient is NHB, ${Hispanic}_{i}$ is similarly an indicator variable for whether the patient is Hispanic, and the omitted category is here NHW. The other variables are defined as in Eq. (1). Both sets of analyses are performed using Stata v.16, and in particular, the built-in survey commands for mean estimates and pooled logistic regression analysis. Model goodness of fit was assessed using model F-tests, and results robustness was also assessed using an alternative probit estimation approach. Statistical significance is noted at levels of *p* < 0.01, *p* < 0.05, and *p* < 0.1 throughout the analyses.

### Sensitivity analysis

While our study design is set up to account for selection on observable characteristics, one potential limitation pertains to the possibility that patients select into our study sample on the basis of unobserved characteristics. In order to examine the sensitivity of our results to this possibility, we estimate a probit model with adjustment for endogenous medical visit selection. Here, our exclusion restriction pertains to patients’ self-reported travel time to their usual and customary source of care. This exclusion restriction assumes that patients’ travel time may influence the propensity with which they seek/receive care (i.e. their selection decision), but that this travel time is not associated with patients’ risk of CHE conditional on having received care (i.e. our outcome model).

## Results

### Sample descriptives and time trends

**Fig. 1:**
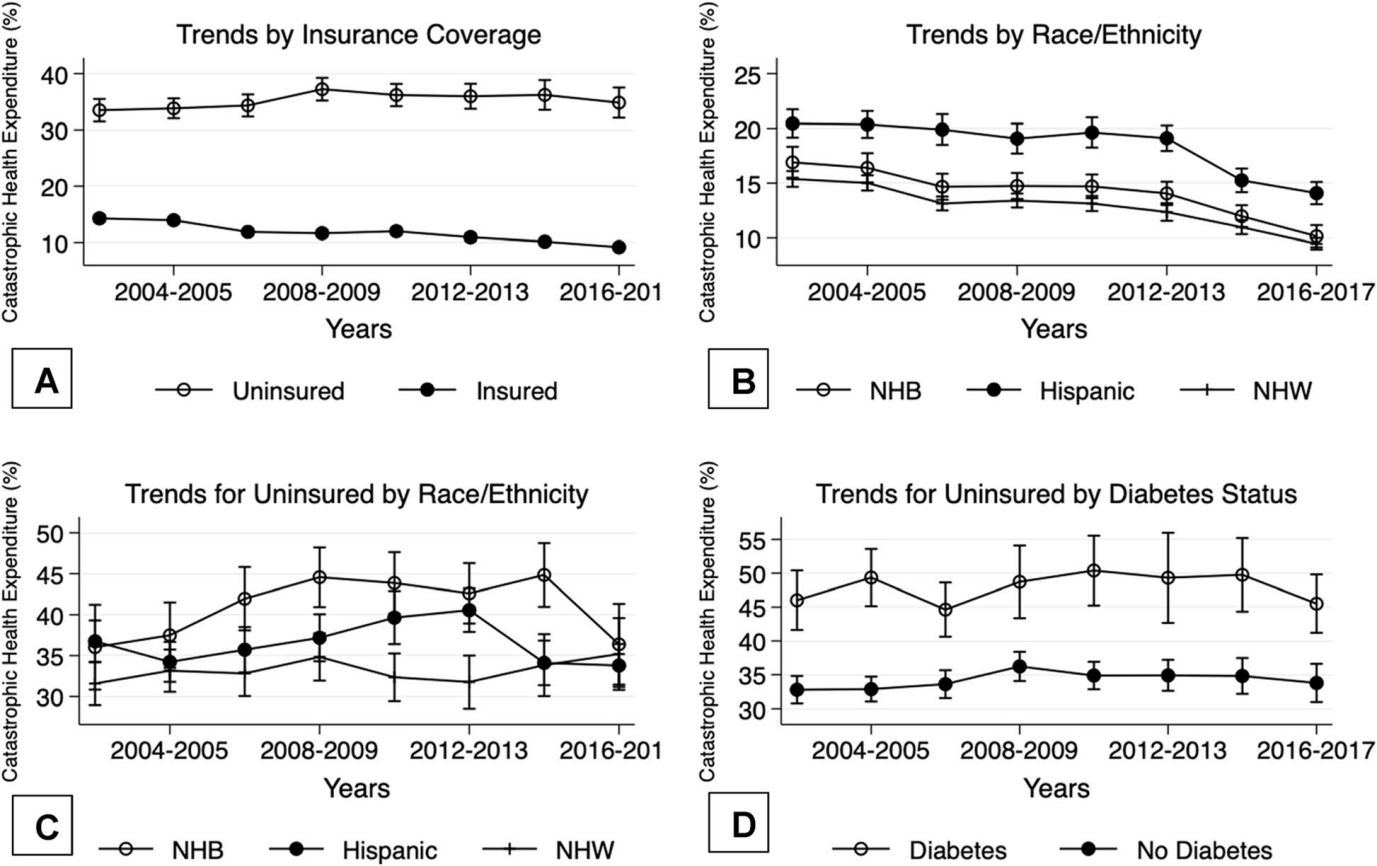
Trends in catastrophic health expenditure incidence based on inflation adjusted ($2017) charges incomes, 2002 – 2017. **A** depicts CHE trends by insurance status.** B** outlines CHE trends by race/ethnicity. **C** and** D** both condition on the uninsured population, and then present trends CHE by race/ethnicity (**C**) and by Diabetes Diagnosis (**D**). All Figures also depict 95% confidence intervals based on linearized standard errors

Table 1 provides summary statistics across the full sample (columns 2,3), the subsample of uninsured patients by race/ethnicity (columns 4, 5, 6), and the subsample of patients with a diabetes diagnosis across race/ethnicity (columns 7, 8, 9). Looking at CHE, we note an overall incidence of 14%, with considerably higher levels for patients that are uninsured (*p* < 0.0001) or have a diabetes diagnosis (*p* < 0.0001). Looking at Fig. 1 (A and D) the CHE gap between insured and uninsured patients, as well as patients with and without a diabetes diagnosis, remained consistent and significant (*p* < 0.05), across the 2002 – 2017 period. Additionally, we note CHE gaps across race/ethnicity both within the full, as well as the uninsured populations, although the significance of these mean differences varied across years (Figs. 1B and C).

**Table 1:** Sample demographics by race/ethnicity, uninsured status and diabetes diagnosis, 2002–2017

	**Full Sample**		**Uninsured**			**Diabetes**		
	**All**		**NHW**	**NHB**	**Hisp**	**NHW**	**NHB**	**Hisp**
	**Mean**	**Lin. SE**	**Mean**	**Mean**	**Mean**	**Mean**	**Mean**	**Mean**
CHE (%)	13.8%	0.1%	33.1%	41.3%	36.6%	17.7%	19.8%	23.5%
Hispanic (%)	11.9%	0.4%	-	-	-	-	-	-
NHB (%)	10.7%	0.4%	-	-	-	-	-	-
Diabetes (%)	10.5%	0.1%	6.0%	9.3%	8.8%	-	-	-
Uninsured (%)	8.8%	0.1%	-	-	-	4.3%	6.2%	14.3%
***Charges & Expenditures***								
Tot. Charges	$12,015.6	$145.0	$5,547.3	$6,228.7	$4,112.6	$26,504.3	$27,713.0	$23,393.5
Tot. Expenditures	$6,455.4	$53.9	$2,827.6	$2,514.7	$1,759.7	$14,637.9	$13,206.2	$10,718.1
***Predisposing Factors***								
Age	48.0	0.1	39.8	39.1	37.5	62.1	58.6	57.2
Female (%)	55.2%	0.1%	49.4%	52.7%	54.6%	49.0%	59.0%	54.4%
Married (%)	54.6%	0.3%	39.9%	23.6%	48.5%	61.3%	39.0%	56.5%
Family Size	2.7	0.0	2.6	2.7	3.6	2.2	2.3	2.9
***Enabling Factors***								
College Degree (%)	30.3%	0.4%	15.4%	9.1%	6.7%	20.4%	13.7%	7.3%
Employed (%)	61.7%	0.3%	64.8%	59.1%	62.7%	39.0%	37.6%	40.2%
***Need Factors***								
High Blood Pressure (%)	35.0%	0.2%	24.6%	35.8%	20.1%	75.3%	83.6%	70.2%
Coronary Heart Disease (%)	5.8%	0.1%	2.6%	1.9%	2.3%	19.8%	14.5%	14.5%
Stroke Diagnosis (%)	4.1%	0.1%	1.9%	3.0%	1.0%	12.1%	13.6%	8.7%
Emphysema Diagnosis (%)	2.4%	0.1%	2.1%	0.7%	0.3%	6.9%	2.8%	2.3%
Arthritis Diagnosis (%)	28.3%	0.2%	20.6%	19.2%	9.0%	55.1%	51.5%	40.8%
ADL Help Needed (%)	2.0%	0.0%	0.7%	0.7%	0.6%	5.6%	7.1%	6.0%
IADL Help Needed (%)	4.0%	0.1%	1.9%	1.5%	0.9%	10.4%	12.7%	10.1%
Self Health (% poor/fair)	15.4%	0.2%	17.4%	21.1%	21.3%	36.9%	42.9%	48.4%
Self Mental (% poor/fair)	7.7%	0.1%	10.1%	9.8%	7.1%	13.7%	16.7%	18.1%
***Medical Events***								
OB Visits	7.3	0.1	4.3	2.9	2.7	12.4	10.6	9.5
OP Visits	0.7	0.0	0.3	0.3	0.2	1.4	1.2	0.8
ER Visits	0.2	0.0	0.3	0.5	0.2	0.4	0.4	0.3
IP Discharges	0.1	0.0	0.1	0.1	0.1	0.3	0.3	0.2
**N**	256,280		10,097	4,894	13,583	13,488	7,384	7,834
**Annual Population Size**	161.2mil		7.5mil	1.8mil	4.0mil	10.6mil	2.6mil	2.4mil

Summary statistics are all based on survey weight adjusted mean estimates. For the full sample we also report linearized standard errors

*ADL* Activities of Daily Living, *CHE* Catastrophic Health Expenditures, *CPI* Consumer Price Index, *IADL* Instrumental Activities of Daily Living, *NHB* Non-Hispanic Black, *NHW* Non-Hispanic White, *OB* Office Based, *OP* Outpatient, *ER* Emergency Room, *IP* Inpatient

Table 1 further indicates that among the population of the uninsured, NHB patients have a significantly higher risk of CHE when compared to NHW (*p* < 0.0001) (or Hispanic, *p* = 0.0002) patients. Figure 1C further indicates that the trends within the CHE gap (between NHB and the NHW/Hispanic patients) appear to have been particularly elevated between the years of 2004 – 2015.

Table 1 also reports mean CHE across race/ethnicity for patients with a diabetes diagnosis. Differences across these means are found to be significant, with NHB patients having a higher risk of CHE than NHW (*p* = 0.016), and Hispanic patients having a higher CHE risk than both NHB (*p* = 0.0003) and NHW (*p* < 0.0001) patients.

### Logistic regression analysis

## Disparity gaps across insurance coverage and diabetes diagnosis

**Fig. 2:**
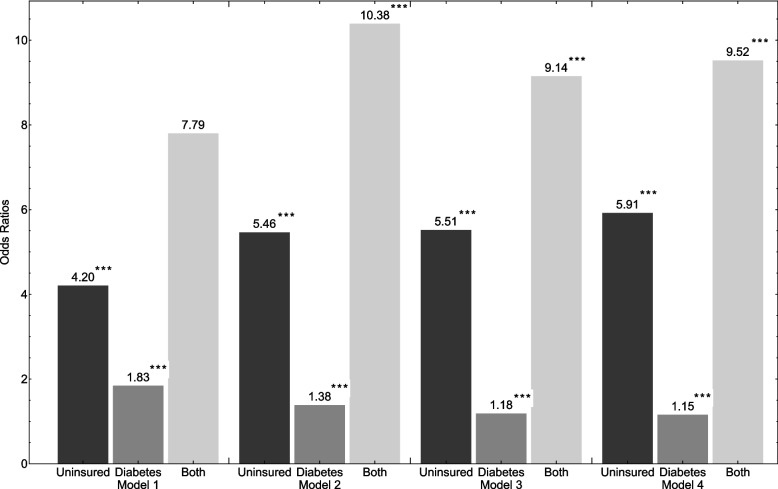
Logistic odds ratios. Here we report the odds ratios across four models – model 1 includes predisposing controls; model 2 includes predisposing and enabling controls; model 3 includes predisposing, enabling and need controls; and model 4 contains predisposing, enabling, need, and medical event controls. All models also contain region and year indicators. The omitted reference category is that of insured patients without a diabetes diagnosis; and significance denoted as: *** *p* < 0.01, ** *p* < 0.05, * *p* < 0.1

Figure 2 shows that our fully adjusted model (which controls for predisposing, enabling and need factors, type of medical event, year and region indicators) indicates that the relative odds of having CHE if uninsured is 5.9 (*p* < 0.01) compared to if insured, and 1.1 (*p* < 0.01) for patients with a diabetes diagnosis (compared to those with no diabetes diagnosis). Interestingly, we further find that there exists a significant (and meaningful) interaction between insurance status and diabetes diagnosis. This interaction indicates that patients who are uninsured, and also have a diabetes diagnosis, are 9.5 (*p* < 0.01) times more likely to experience CHE than insured patients without a diabetes diagnosis. As such, we note the total effect of being uninsured and having a diabetes diagnosis on CHE risk far exceeds the sum total risk of each of these effects individually.

## Disparity gaps across race/ethnicity within the vulnerable patient populations that are uninsured and/or have a diabetes diagnosis

Table 2 allows us to explore disparity gaps across race/ethnicity within the patient populations of those that are uninsured, have a diabetes diagnosis, or both – that is, within populations identified to be at elevated risk of CHE. Looking at the bottom section of Table 2, we see the results from the fully adjusted model (Model 4). Firstly, for the subpopulation of uninsured we note significant disparities across race/ethnicity. Here,

**Table 2:** Logistic odds ratio regression estimates from stratified subsample analysis

**Sample/Subsample:**	(1)	(2)	(3)
	Uninsured	Diabetes	Both
	Pr(CHE)	Pr(CHE)	Pr(CHE)
***Model 1 – Predisposing Controls***			
NHB	1.26***	1.08	0.96
	(0.06)	(0.06)	(0.16)
Hispanic	0.98	1.19***	0.99
	(0.04)	(0.06)	(0.12)
Uninsured		4.77***	
		(0.33)	
Diabetes	1.85***		
	(0.11)		
***Model 2 – Predisposing and Enabling Controls***			
NHB	1.21***	0.99	0.94
	(0.07)	(0.06)	(0.18)
Hispanic	0.98	1.09	1.06
	(0.05)	(0.06)	(0.17)
Uninsured		6.68***	
		(0.58)	
Diabetes	1.59***		
	(0.11)		
***Model 3 – Predisposing, Enabling and Need Controls***			
NHB	1.23***	0.96	0.99
	(0.07)	(0.06)	(0.18)
Hispanic	1.02	1.07	1.14
	(0.05)	(0.06)	(0.18)
Uninsured		6.87***	
		(0.59)	
Diabetes	1.26***		
	(0.09)		
***Model 4 – Predisposing, Enabling, Need and Event Controls***			
NHB	1.13**	0.97	1.01
	(0.06)	(0.06)	(0.20)
Hispanic	1.14**	1.09	1.39**
	(0.06)	(0.06)	(0.23)
Uninsured		7.18***	
		(0.61)	
Diabetes	1.15		
	(0.11)		
***All Models***			
Observations	30,280	31,073	2,675
Year Indicators	X	X	X
Region Indicators	X	X	X

Standard Errors are reported in parentheses. Odds Ratios are reported with significance denoted as: *** *p* < 0.01, ** *p* < 0.05. NHW is the omitted reference category for race/ethnicity. Uninsured refers to the subsample of patients that are uninsured; Diabetes refers to the subsample of patients with a diabetes diagnosis; Both is the sample of patients that are both uninsured and have a diabetes diagnosis. The model heading describes the controls included within the model specification, and all specifications additionally include controls for year and region indicators

NHBs have 13% (*p* < 0.05), and Hispanics 14.2% (*p* < 0.05), higher odds of experiencing CHE than NHWs. Second, looking at the diabetes diagnosis subsample, we do not note any significant differences, however, when we consider the subsample of patients that are both uninsured and have a diabetes diagnosis we find that Hispanic patients have 39.3% higher relative odds of CHE than do NHW patients. This finding, however, appears sensitive to the model specification (within Table 2), as it holds for our preferred model specification (Model 4), but not consistently across other specifications. Lastly, we note that these results do not appear to be driven by our decision of employing a Logistic model estimation strategy as results based on Probit model estimates reveal qualitatively similar findings (see Additional file 1: Appendix B).

## Sensitivity analysis

As noted within the methods section, to examine the sensitivity of our racial/ethnic disparity gap estimates to the possibility of patients’ selecting into our sample on unobservable characteristics, we also estimate a structural model that accounts for endogenous sample selection – one utilizing an exclusion restriction for patients’ self-reported travel time to their usual and customary care. This yield results that were qualitatively similar to our main results, indicating significant disparity gaps in CHE across race/ethnicity. These results are available upon request.

## Limitations

We note a number of limitations of our study. First, it is important to recognize that our results are based on an observational study design and as such these results should be interpreted as associations and not as causal relationships. Second, our study focuses on the adult US population who had at least one medical event, and as such, our results may not generalize beyond this population. Third, the logit estimation approach employed within this study relies on four primary assumptions – the independence of observations; that continuous predictors are linearly related to the transformed version of the outcome; that errors be logistically distributed; and that there not be perfect multicollinearity among independent variables [24]. Here, we note that results based on a probit model, which instead assumes normally distributed errors, yield qualitatively similar results (please see our robustness test results within the Additional file 1: Appendix B). Additionally, while multicollinearity between some of our covariates could be a concern, correlation patterns among our variables suggests that this is not a concern within our application (additional correlation tables are provided within the Additional file 1: Appendix A).

## Discussion

This study has two main findings. The first is that CHE risks increase dramatically with lack of insurance coverage and with a diabetes diagnosis. Additionally, the findings here indicate that there exists a significant interaction between having a diabetes diagnosis and being uninsured. This finding implies that the total CHE risk for patients that are both uninsured and have a diabetes diagnosis is larger than the sum total of the CHE risk for patients that are either uninsured or have a diabetes diagnosis. As such, this finding identifies a particularly vulnerable population from the perspective of CHE risk. This finding thus adds uninsured patients with diabetes to the list of particularly vulnerable patient populations, where other work within the US context has previously identified low-income adults with atherosclerotic cardiovascular disease, and uninsured trauma patients, as having a high risk of CHE [30, 46]. These patient populations all represent populations where tailored financial support may be called for in order to help ameliorate noted CHE risks. The second, and perhaps most significant finding of this study pertains to racial/ethnic disparity gaps within the uninsured patient population, and the patient population that is both uninsured and has a diabetes diagnosis. Given that race/ethnicity is a social construct without any real physiological basis [18, 21, 52], it is important to consider where these disparity gaps may emanate from – especially given the rich set of control variables utilized within this study.

One explanation is that these gaps represent different levels in exposure to structural racism (SR) across the race/ethnicity populations considered by this study. Such exposure may cause disparity gaps in CHE across race/ethnicity in a number of ways. First, it may affect the propensity for CHE by means of affecting individuals’ education, employment, and in turn income – causing individuals with SR exposure to have added sensitivity towards high medical charges. Second, SR may increase the risk of CHE by means of increasing the likelihood that racial/ethnic minorities are uninsured or underinsured, something that puts them at an elevated risk of being billed inflated chargemaster prices when they do seek care. This observation draws on early reports indicating that individuals residing within areas with exposure to historic structural racist laws, who tend to be racial/ethnic minorities, to this day experience medical access disadvantages [32, 37, 38]. Third, insurance access constraints may further affect where these patients are able to seek care, and the type of care that they are able to receive. Access constraints of this nature may further exacerbate difficulties related to the already difficult task of trying to price-shop for low-cost care in a market that at large lacks full (or at least consumer friendly) transparency pertaining to price and charges [43]. All of these factors may act to create a linkage between historical structural racism, and present-day risk of exposure to CHE. A particularly concerning observation here, however, is that this linkage risks perpetuating longstanding structural inequities by putting racial/ethnic minorities at increased risk of having to face the potential consequences of high CHE risk – that is, the elevated risk of financial hardship, and loss of credit access, which in turn restricts individuals’ ability to build intergenerational wealth. As such, disparities in CHE risk may perpetuate structural inequities that were historically instituted by means of racist laws and policies within the US [11, 12, 20], and which recent work has shown remain adversely associated with present day health outcomes [34, 36].

Policy solutions are thus needed to help reduce the elevated CHE risk experienced by racial/ethnic groups. First, there is a need for policies that can help ensure chargemaster price growth is limited. Such limits may be explicitly legislated via price regulations [45], but they may also be encouraged by support of efforts seeking to ensure greater price transparency within the US health care system. While such efforts are currently underway in the form of public price reporting requirements (under the Health Services Act), as well as requirements on the provision of good-faith estimates to self-pay patients (under the recent No Surprises Act) (CMS 2022 [17]), more work is needed to ensure broad provider compliance with existing laws (Ayoub and Balakrishnan 2022 [8]), and to ensure consumers can access provider price disclosures in an accessible and easy to understand way [35], and to ensure that price disclosures do not unintentionally lead patients to increasingly forgo important care. Second, some scholars have also suggested that the US health care market needs to adopt an implied-contracts approach for the settlement of health care payments [44, 45]. As noted by [45], such an approach could ensure patients are only asked to “pay whatever amount a prudent patient and provider would have agreed to, given appropriate time and information”. Naturally, such an approach would require adequate oversight and that patients be aware of what might constitute appropriate billing for a given service. While this might be too much to expect from patients, alternative legal support could be extended to patients in cases of CHE events in the form of accessible public arbitration services. These provisions could come in the form of extensions of existing law that extends such services to insurers under the recent No Surprises Act in the US (CMS 2022 [17]). Beyond policies that directly target pricing or the settlement of payment disputes within the US health care market, there is also a need for policies that help ensure patient protection from receipt of inflated chargemaster bills by means of insurance coverage. Here, the ensuring of Medicaid expansion adoption within current non-expansion states could be an important policy tool for accomplishing this goal [5, 6].

Our hope is that this work can help spur policy consideration as well as further work within this area. Regarding future research, there appears to be several avenues for such efforts. Firstly, work examining the extent to which our results are potentially downward biased due to selection on the basis of unobserved anticipated CHE risk presents an important extension. Second, extensions of our analysis to the study of CHE among other patient populations in terms of other health conditions, as well as extensions to study potential disparities in CHE across sex, appear warranted. Lastly, we need further work that can help explore the potential linkage between structural racism, present day risk of CHE, and subsequent economic and health disparities.

## Conclusion

In conclusion, our study has several findings. First, we find that uninsured patients have significantly higher risk of CHE than insured patients. This finding highlights the importance of continued efforts towards ensuring broad based insurance coverage within the US healthcare system. These efforts appear particularly important given the recent trends in uninsurance rate increases within the US. Second, our findings indicate that patients with a diabetes diagnosis are at a significantly elevated risk of experiencing CHE compared to patients without such a diagnosis. This finding highlights the importance of ensuring affordability of medical care for individuals living with a chronic condition such as diabetes. Third, our study also highlights that uninsured patients with diabetes are at a significantly elevated risks for CHE. We note that the elevation of risk for these patients is in excess of that of individuals who are only uninsured or only have a diabetes diagnosis. As such, these findings highlight uninsured individuals with a diabetes diagnosis as a highlight vulnerable population, with a particularly high risk of experiencing CHEs.

Lastly, our findings indicate that compared to NHW individuals, the risks of CHEs are further disproportionately higher among uninsured NHB and Hispanic individuals. These results are important to emphasize as they suggest that CHE may represent a channel through which structural economic and health disparities are perpetuated within the US health care system.

### Supplementary Information

**Additional file 1:** **Appendix A.** Additional Summary Descriptives -- Pairwise Correlation Tables Across Analyses Samples. **Table S1.** Pairwise Correlations. **B**. Estimation Method Robustness Check -- Estimates based on Probit Model.

## Data Availability

The datasets used and/or analyzed during the current study are available from the corresponding author on reasonable request.

## Abbreviations

ADL: Activities of Daily Living
CHE: Catastrophic Health Expenditures
CPI: Consumer Price Index
IADL: Instrumental Activities of Daily Living
MEPS: Medical Expenditure Panel Survey
NHB: Non-Hispanic Black
NHW: Non-Hispanic White
