# Supplementary material for: Catastrophic health expenditures: a disproportionate risk in uninsured ethnic minorities with diabetes
Source: Health Econ Rev. 2024 Mar 6;14:18. doi: 10.1186/s13561-024-00486-7 (PMC10916057; doi:10.1186/s13561-024-00486-7)
Supplement: Supplementary file 1 — Additional file 1: Appendix A. Additional Summary Descriptives -- Pairwise Correlation Tables Across Analyses Samples. Table S1. Pairwise Correlations. B. Estimation Method Robustness Check -- Estimates based on Probit Model. [file 13561_2024_486_MOESM1_ESM.docx]

**Supplemental Appendix**

**A. Additional Summary Descriptives -- Pairwise Correlation Tables Across Analyses Samples.**

Covariates based on the Andersen and Nyman health care utilization framework contain variables that are likely to be correlated. If correlations are high, this might impact the precision of estimates, however, as Table S1 shows (see below), the pairwise correlation patterns among potentially sensitive variables are rather moderate and therefore unlikely to present an issue to our analyses.

**Table S1: Pairwise Correlations**

| Variables | (1) | (2) | (3) | (4) | (5) | (6) | (7) | (8) |  |
| --- | --- | --- | --- | --- | --- | --- | --- | --- | --- |
| (1) Age | 1.000 |  |  |  |  |  |  |  |  |
| (2) College Degree | 0.008 | 1.000 |  |  |  |  |  |  |  |
| (3) Employed | -0.332 | 0.193 | 1.000 |  |  |  |  |  |  |
| (4) Tot. Personal Income | 0.055 | 0.426 | 0.421 | 1.000 |  |  |  |  |  |
| (5) Uninsured | -0.171 | -0.128 | 0.012 | -0.151 | 1.000 |  |  |  |  |
| (6) High Blood Pressure | 0.476 | -0.093 | -0.229 | -0.052 | -0.081 | 1.000 |  |  |  |
| (7) Coronary Heart  Disease | 0.264 | -0.043 | -0.176 | -0.047 | -0.052 | 0.232 | 1.000 |  |  |
| (8) Stroke Diagnosis | 0.213 | -0.053 | -0.168 | -0.065 | -0.046 | 0.188 | 0.196 | 1.000 |  |
|  | | | | | | | | | |

**B. Estimation Method Robustness Check -- Estimates based on Probit Model.**

Given that our outcome measure (CHE) is a binary measure, we employed a logistic regression model estimation approach within the main text. An alternative modelling approach would have been the use of a probit model, however, results based on this model results in qualitatively very similar results (see Table S2 below, however, note that these are probit regression estimates rather than odds ratios). As such, we note that our findings do not appear to be sensitive to this modelling choice.

**Table S2:** Probit Regression Estimates from Stratified Subsample Analysis.

|  | (1) | (2) | (3) |
| --- | --- | --- | --- |
| **Sample/Subsample:** | Uninsured | Diabetes | Both |
|  | Pr(CHE) | Pr(CHE) | Pr(CHE) |
| ***Model 1 – Predisposing Controls*** | | | |
| NHB | 1.153*** | 1.045 | 0.978 |
|  | (0.033) | (0.032) | (0.097) |
| Hispanic | 0.983 | 1.106*** | 0.993 |
|  | (0.025) | (0.033) | (0.078) |
| Uninsured |  | 2.526*** |  |
|  |  | (0.104) |  |
| Diabetes | 1.458*** |  |  |
|  | (0.054) |  |  |
| ***Model 2 – Predisposing and Enabling Controls*** | | | |
| NHB | 1.117*** | 0.992 | 0.970 |
|  | (0.035) | (0.034) | (0.105) |
| Hispanic | 0.985 | 1.043 | 1.038 |
|  | (0.026) | (0.034) | (0.093) |
| Uninsured |  | 2.909*** |  |
|  |  | (0.134) |  |
| Diabetes | 1.306*** |  |  |
|  | (0.053) |  |  |
| ***Model 3 – Predisposing, Enabling and Need Controls*** | | | |
| NHB | 1.126*** | 0.975 | 0.995 |
|  | (0.034) | (0.034) | (0.105) |
| Hispanic | 1.010 | 1.037 | 1.081 |
|  | (0.027) | (0.035) | (0.095) |
| Uninsured |  | 2.956*** |  |
|  |  | (0.136) |  |
| Diabetes | 1.139*** |  |  |
|  | (0.048) |  |  |
| ***Model 4 – Predisposing, Enabling, Need and Event Controls*** | | | |
| NHB | 1.073** | 0.982 | 1.017 |
|  | (0.035) | (0.034) | (0.114) |
| Hispanic | 1.078** | 1.050 | 1.197* |
|  | (0.032) | (0.036) | (0.110) |
| Uninsured |  | 3.036*** |  |
|  |  | (0.137) |  |
| Diabetes | 1.085 |  |  |
|  | (0.055) |  |  |
| ***All Models*** |  |  |  |
| Observations | 30,280 | 31,073 | 2,675 |
| Year Indicators | X | X | X |
| Region Indicators | X | X | X |

Standard Errors are reported in parentheses. Point estimates are reported with significance denoted as: *** p<0.01, ** p<0.05, * p<0.1. NHW is the omitted reference category for race/ethnicity. Uninsured refers to the subsample of patients that are uninsured; Diabetes refers to the subsample of patients with a diabetes diagnosis; Both is the sample of patients that are both uninsured and have a diabetes diagnosis. The model heading describes the controls included within the model specification, and all specifications additionally include controls for year and region indicators.
